# Association between the FT3/FT4 ratio and in-hospital mortality among hospitalized patients undergoing thyroid function testing

**DOI:** 10.3389/fmed.2026.1792939

**Published:** 2026-10-27

**Authors:** Min Peng, Jia Wang, Jinming Yu, Lijie Ren, Gelin Xu

**Affiliations:** 1 Department of Neurology, Institute of Translational Medicine, The First Affiliated Hospital of Shenzhen University, Shenzhen Second People's Hospital, Shenzhen, China; 2 Department of Otolaryngology, Institute of Translational Medicine, The First Affiliated Hospital of Shenzhen University, Shenzhen Second People's Hospital, Shenzhen, China

**Keywords:** all-cause mortality, association, cohort, FT3/FT4 ratio, thyroid hormones

## Abstract

**Background:**

Thyroid function is highly sensitive to acute illness and systemic stress in hospitalized patients, and the free triiodothyronine/free thyroxine (FT3/FT4) ratio has been proposed as an integrative marker reflecting peripheral thyroid hormone metabolism. This study aimed to investigate the associations between thyroid function parameters, particularly the FT3/FT4 ratio, and in-hospital all-cause mortality.

**Methods:**

A total of 26,223 inpatients from all departments of a large tertiary hospital in China were included. Serum thyroid-stimulating hormone (TSH), FT3, FT4, and the FT3/FT4 ratio (×100) were measured at admission, and in-hospital all-cause mortality was identified from electronic medical records. Cox proportional hazards regression models were used to assess associations between thyroid hormone parameters and mortality. Nonlinear associations were evaluated using restricted cubic spline analyses. Sensitivity analyses included exclusion of extreme thyroid hormone values, inverse-probability-of-selection weighting using the entire source population, and stratified Cox regression to address non-proportional hazards. Subgroup and exploratory analyses of admission vital signs were also performed.

**Results:**

Higher TSH (HR = 1.015, 95% CI: 1.010–1.020) and FT4 (HR = 1.017, 95% CI: 1.002, 1.033) were associated with higher in-hospital mortality, whereas higher FT3 (HR = 0.865, 95% CI: 0.806, 0.929) and FT3/FT4 ratio (×100) (HR = 0.974, 95% CI: 0.964, 0.983) were associated with lower mortality. RCS analyses showed evidence of nonlinearity for TSH, but not for FT3, FT4, or the FT3/FT4 ratio. The inverse association for the FT3/FT4 ratio remained in the IPW analysis (HR = 0.954, 95% CI: 0.945, 0.963) and in the Cox model stratified to address non-proportional hazards (HR = 0.970, 95% CI: 0.961, 0.980). Sensitivity analyses excluding extreme values generally supported the primary findings, particularly for FT3 and the FT3/FT4 ratio. Subgroup analyses suggested potential heterogeneity across several patient characteristics. Exploratory analyses showed associations between the FT3/FT4 ratio and admission vital signs, whereas only temperature was independently associated with mortality after multivariable adjustment.

**Conclusion:**

The FT3/FT4 ratio was independently associated with in-hospital mortality and may serve as a potential indicator of outcomes among patients undergoing thyroid function testing.

## 1 Introduction

Major causes of in-hospital mortality include pneumonia, cerebrovascular events, myocardial infarction, diabetes, and cancer (1). Established risk factors for mortality and prolonged hospitalization include advanced age, cognitive impairment, malnutrition, multiple comorbidities, and frailty (2). However, the clinical utility of circulating biomarkers for risk stratification in hospitalized patients remains incompletely understood.

Thyroid hormones (THs), including free triiodothyronine (FT3) and free thyroxine (FT4), play essential roles in maintaining physiological homeostasis. THs regulate growth, development, and energy metabolism throughout life, beginning from early developmental stage (3, 4). Dysregulation of thyroid hormone metabolism not only results in thyroid disorders, such as hyperthyroidism and hypothyroidism, but is also closely associated with the development and progression of various chronic diseases through effects on metabolism, inflammation, and immune regulation (5).

Previous studies have explored the associations between the FT3/FT4 ratio and mortality outcomes, although most were based on population databases, particularly the National Health and Nutrition Examination Survey (NHANES). The FT3/FT4 ratio has been reported to be associated with all-cause mortality in the general U.S. population (6). Notably, a U-shaped association between the FT3/FT4 ratio and mortality has been observed in community populations (7), whereas a J-shaped association with 5-year cardiovascular mortality has been reported among patients with chronic kidney disease (CKD) (8). Additionally, lower FT3/FT4 ratios have been associated with elevated cardiovascular mortality in patients with CKD (9, 10). Separately, FT3 levels have generally shown inverse associations with all-cause mortality, whereas FT4 levels have demonstrated positive associations (6, 11). One study further suggested that low-normal thyroid-stimulating hormone (TSH) levels, rather than FT3 or FT4 concentrations, were independently associated with all-cause mortality among euthyroid older adults (12).

Despite these findings, several important knowledge gaps remain. Most previous studies were conducted in community-based populations or specific disease cohorts, predominantly among Western populations, and their findings may not be directly applicable to hospitalized patients, in whom TH metabolism is substantially affected by acute illness and systemic stress. Moreover, the relative prognostic value of TSH, FT3, FT4, and the FT3/FT4 ratio remains inconsistent across studies, suggesting potential context-dependent associations. Therefore, further investigation is needed to clarify the relationships between different thyroid function parameters and mortality risk in hospitalized populations, particularly in non-Western settings.

In this retrospective cohort study, we aimed to investigate the associations between thyroid function parameters, including TSH, FT3, FT4, and the FT3/FT4 ratio, and in-hospital all-cause mortality among hospitalized patients undergoing thyroid function testing in China.

## 2 Methods

### 2.1 Study population

We identified hospitalized patients from the electronic medical records database of Shenzhen Second People's Hospital between October 2013 and March 2025. For patients with multiple hospitalization records during the study period, only the first hospitalization record was retained, resulting in 66,210 unique patients eligible for subsequent screening. Patients with incomplete thyroid function measurements (missing TSH, FT3, or FT4 values) were first excluded (*n* = 39,983). Among the remaining patients, 4 individuals with documented previous or current thyroid disorders were further excluded. These disorders included hyperthyroidism, hypothyroidism, Hashimoto's thyroiditis, diffuse goiter, toxic nodular goiter, and other thyroid diseases that could affect thyroid function tests. Of the 26,223 patients included in the study cohort, 2 had unavailable discharge dates and consequently missing survival times; these patients were excluded from the Cox regression analyses. (Figure 1).

**Figure 1:**
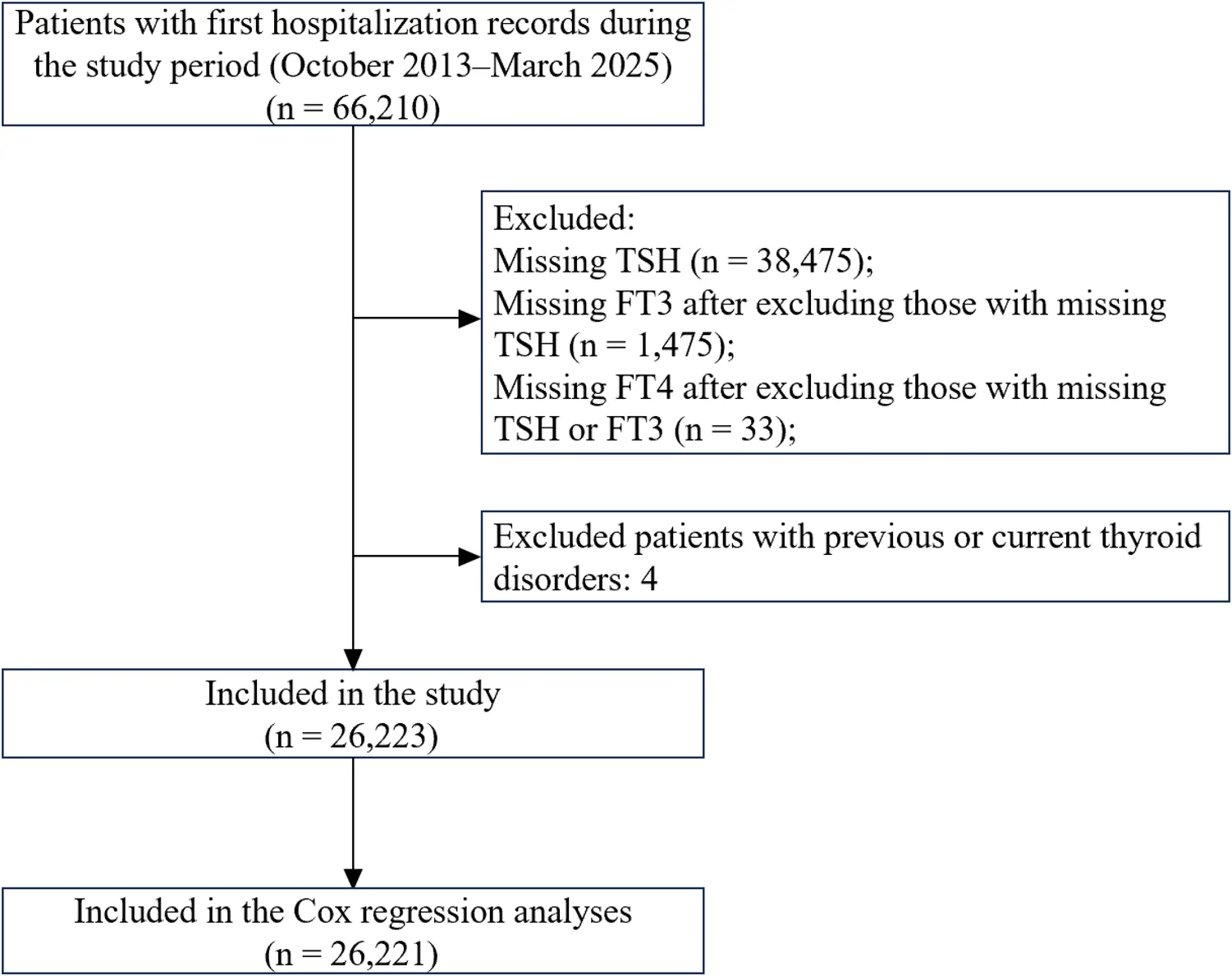
Flow chat of this study.

The study protocol was approved by the Institutional Review Board (IRB) of Shenzhen Second People's Hospital. The requirement for informed consent was waived because of the retrospective study design and the use of de-identified patient data.

### 2.2 Data collection

The following variables were collected: age, sex, marital status, medical insurance category, admission method, admission body temperature, admission heart rate, admission systolic blood pressure, admission diastolic blood pressure, admission time period, medical history (including hypertension, coronary heart disease, and diabetes mellitus), smoking and drinking history, primary admission diagnoses, and ICU admission status. Primary admission diagnoses were categorized into major disease groups, including cancer, infection/sepsis, stroke, heart failure, chronic kidney disease, and liver disease. Because direct ICU admission information was unavailable, discharge from intensive care-related departments (including EICU, MICU, SICU, NsICU, ICU, CCM, and NCCU) was used as a proxy indicator of critical illness.

### 2.3 Thyroid hormones

Blood samples were collected at 5:00 a.m. after an overnight fast. Thyroid function parameters, including TSH, FT3, and FT4, were obtained from laboratory tests results and measured using chemiluminescence immunoassay (Siemens, USA). The reference intervals were as follows: FT3, 2.76−6.45 pmol/L; FT4, 11.20–23.81 pmol/L; TSH, 0.35–5.10 mIU/mL. Thyroid function tests were not routinely performed in all hospitalized patients but were ordered according to clinical indications. Testing was performed for patients with severe illness, those screened for high-risk factors of cardiovascular and cerebrovascular diseases, as well as patients requiring thyroid assessment due to other concurrent endocrine comorbidities.

### 2.4 Outcome

The primary outcome was in-hospital all-cause mortality, defined as death from any cause occurring during the index hospitalization. The observation period began on the date of hospital admission and ended at in-hospital death or hospital discharge, whichever occurred first. Survival time was calculated in days from the date of admission to the date of in-hospital death or discharge. Patients discharged alive were censored at the date of discharge. Patients discharged alive were considered censored observations at the time of discharge.

### 2.5 Statistical analysis

Continuous variables were assessed for distributional characteristics. Because the variables did not follow normal distributions, non-parametric tests were used. Categorical variables were compared using the chi-square test.

Associations between continuous thyroid function parameters (TSH, FT3, FT4, and FT3/FT4 ratio) and in-hospital mortality were initially evaluated using Cox proportional hazards regression models. The FT3/FT4 ratio was categorized into tertiles for additional analyses. For ease of interpretation, the FT3/FT4 ratio was multiplied by 100 before regression analyses; therefore, the estimated hazard ratio represents the effect per 0.01-unit increase in the original FT3/FT4 ratio.

Three Cox proportional hazards models were constructed to estimate hazard ratios (HRs) and 95% confidence intervals (CIs). The crude model included no adjustment covariates. Model 1 was adjusted for age and sex. Model 2 was additionally adjusted for marital status, admission approach, admission time period, admission vital signs (temperature, heart rate, systolic blood pressure, and diastolic blood pressure), hypertension, coronary heart disease, diabetes mellitus, cancer, infection, stroke, heart failure, kidney disease, liver disease, ICU admission, smoking, and drinking. Covariates were selected *a priori* based on clinical plausibility and previously reported associations with mortality outcomes to minimize confounding bias. Some potential confounders, including detailed clinical severity scores, laboratory indicators, nutritional status, and medication use, were unavailable or had substantial missing data and therefore could not be included in the primary analyses.

The proportional hazards assumption was assessed using Schoenfeld residuals. Because the global test indicated evidence of nonproportional hazards in the primary model, we conducted a sensitivity analysis using a stratified Cox model, allowing separate baseline hazards across age groups (<45, 45–64, and ≥ 65 years), sex, coronary heart disease, and cancer, which showed evidence of nonproportionality in the diagnostic analyses.

Kaplan–Meier curves were generated to illustrate survival probabilities across FT3/FT4 ratio tertiles, and differences were evaluated using the log-rank test. For visual clarity, the Kaplan–Meier curves were displayed through 100 days after admission; patients with longer hospital stays were retained in the survival analysis. Subgroup analyses were performed to assess the consistency of the association between the FT3/FT4 ratio and in-hospital mortality across subgroups defined by age (< 60/ ≥ 60), sex (male/female), marital status (married/divorced/separated), admission approach (outpatient/emergency), admission time period (day/night), hypertension, coronary heart disease, diabetes mellitus, cancer, infection, stroke, heart failure, kidney disease, liver disease, ICU admission, smoking, and drinking. In these analyses, the FT3/FT4 ratio was modeled as a continuous variable (×100). Multivariable Cox models were adjusted for the same covariates as Model 2, except for the variable used to define each subgroup. Effect modification was formally assessed by including an interaction term between the continuous FT3/FT4 ratio and each subgroup variable, with *P* values for interaction derived from likelihood ratio tests comparing models with and without the corresponding interaction term. Subgroup-specific numbers of events and participants were also reported to facilitate assessment of estimate precision.

Restricted cubic spline (RCS) functions with 3 knots placed at fixed percentiles were used within Cox proportional hazards models to flexibly evaluate the shape of the association between thyroid hormone parameters and in-hospital mortality. The knot locations were specified according to Harrell's recommended percentile placement. Hazard ratios were estimated using multivariable Cox regression with adjustment for predefined confounders, and the median value of each exposure variable was used as the reference (TSH, 1.82 mIU/L; FT3, 4.11 pmol/L; FT4, 15.27 pmol/L; and FT3/FT4 ratio ×100, 26.85). Non-linearity was assessed by comparing the full spline model with a corresponding linear Cox model using a likelihood ratio test.

Sensitivity analyses were performed to evaluate the robustness of the findings. For each thyroid hormone parameter, observations below the 5th percentile and above the 95th percentile were excluded, and Cox proportional hazards models were re-estimated using the corresponding restricted datasets.

To further assess the potential impact of selection related to study inclusion, inverse-probability-of-selection weighting (IPW) analyses were performed using the entire source population of 66,210 hospitalized patients. The probability of inclusion in the study cohort was estimated using logistic regression. The primary selection model included age, sex, marital status, admission approach, hypertension, coronary heart disease, diabetes mellitus, cancer, infection, stroke, heart failure, kidney disease, liver disease, ICU admission, smoking, and drinking. Age was missing in 37 patients (0.06%) and was imputed using the cohort median. Stabilized inverse-probability-of-selection weights were calculated and applied to Cox proportional hazards models with robust standard errors. An expanded IPW analysis was additionally performed by incorporating admission temperature, heart rate, systolic blood pressure, and diastolic blood pressure into the selection model. Missing vital-sign values were median-imputed, and corresponding missingness indicators were included in the expanded selection model. Covariate balance before and after weighting was assessed using standardized mean differences (SMDs), with an absolute SMD <0.10 considered indicative of adequate balance.

Exploratory analyses were conducted to further characterize the associations among the FT3/FT4 ratio, admission vital signs, and in-hospital mortality. Linear regression models were used to evaluate associations between the FT3/FT4 ratio and individual admission vital signs. Multivariable Cox proportional hazards models were subsequently applied to examine the associations between each admission vital sign and in-hospital mortality, adjusting for the same covariates as in the primary analyses. Given the observational study design and the near-simultaneous measurement of THs and vital signs at admission, these analyses were considered exploratory and were not intended to establish causal mediation relationships.

Statistical analyses were performed by R 4.2.1 software. Statistical significance was defined as *P* < 0.05.

## 3 Results

### 3.1 Characteristics of patients

A total of 66,210 hospitalized patients were screened, of whom 39,987 patients were excluded. Baseline characteristics between included and excluded participants were compared in Supplementary Table S1. Finally, 26,223 patients were included in the study cohort. The median age of the study population was 67 years, and 50.81% of participants were male (Table 1). Compared with non-survivors, survivors were younger, had a lower proportion of males, higher proportion of outpatient admissions and daytime admissions, and fewer previous hospitalizations (all *P* < 0.05). Regarding vital signs at admission, survivors had lower body temperature and heart rate but higher blood pressure than non-survivors (all *P* < 0.05). Additionally, survivors had lower prevalences of hypertension, coronary heart disease, and diabetes mellitus, but a higher rate of a history of alcohol consumption (all *P* < 0.05).

**Table 1:** The baseline data grouped by death among all patients.

Variables	Overall	Death			
		No	Yes	SMD	*P*
Num.	26,223	25,073	1,150		
Age, years	67.00 [58.00, 75.00]	67.00 [58.00, 75.00]	77.00 [69.00, 83.00]	0.728	<0.001
Sex (Male)	13,323 (50.81)	12,656 (50.48)	667 (58.00)	0.151	<0.001
Marital status				0.217	<0.001
Divorced	731 (2.79)	716 (2.86)	15 (1.30)		
Married	23,487 (89.57)	22,468 (89.61)	1,019 (88.61)		
Separated	1,435 (5.47)	1,330 (5.30)	105 (9.13)		
Single	356 (1.36)	346 (1.38)	10 (0.87)		
Unknown	214 (0.82)	213 (0.85)	1 (0.09)		
Medical Insurance category				0.023	0.473
Urban Resident Insurance	21,046 (80.26)	20,133 (80.30)	913 (79.39)		
Rural Medical Insurance	5,177 (19.74)	4,940 (19.70)	237 (20.61)		
Admission method				0.262	<0.001
Emergency	4,198 (16.01)	3,922 (15.64)	276 (24.00)		
Outpatient	19,673 (75.02)	18,941 (75.54)	732 (63.65)		
Others	2,352 (8.97)	2,210 (8.81)	142 (12.35)		
Admission vital signs					
Body temperature ( °C)^a^	36.50 [36.30, 36.59]	36.50 [36.30, 36.50]	36.50 [36.30, 36.70]	0.044	<0.001
Heart rate (bpm)^b^	77.00 [70.00, 85.00]	77.00 [69.00, 85.00]	80.00 [72.00, 92.00]	0.349	<0.001
Systolic pressure (mmHg)^c^	131.00 [118.00, 147.00]	131.00 [118.00, 147.00]	128.00 [115.00, 148.00]	0.012	0.009
Diastolic pressure (mmHg)^d^	78.00 [70.00, 86.00]	78.00 [70.00, 86.00]	74.00 [66.00, 83.00]	0.132	<0.001
Admission time period				0.153	
Nighttime	1,057 (4.03)	972 (3.88)	85 (7.39)		<0.001
Daytime	25,166 (95.97)	24,101 (96.12)	1,065 (92.61)		
Medical history					
Hypertension	12,666 (48.30)	11,993 (47.83)	673 (58.52)	0.215	<0.001
Coronary heart disease	2,842 (10.84)	2,606 (10.39)	236 (20.52)	0.283	<0.001
Diabetes mellitus	5,911 (22.54)	5,590 (22.29)	321 (27.91)	0.13	<0.001
Cancer	2,127 (8.11)	1,898 (7.57)	229 (19.91)	0.364	<0.001
Infection	1,047 (3.99)	875 (3.49)	172 (14.96)	0.404	<0.001
Stroke	3,326 (12.68)	3263 (13.01)	63 (5.48)	0.262	<0.001
Heart failure	440 (1.68)	366 (1.46)	74 (6.43)	0.258	<0.001
Kidney diseases	1,521 (5.80)	1,373 (5.48)	148 (12.87)	0.258	<0.001
Liver diseases	59 (0.22)	56 (0.22)	3 (0.26)	0.008	1
ICU admission	795 (3.03)	599 (2.39)	196 (17.04)	0.511	<0.001
Smoking ever	6,654 (25.37)	6,375 (25.43)	279 (24.26)	0.027	0.394
Drinking	4,049 (15.44)	3,905 (15.57)	144 (12.52)	0.088	0.006
TSH mIU/L	1.80 [1.10, 2.91]	1.81 [1.11, 2.89]	1.73 [0.77, 3.27]	0.160	0.008
FT3 pmol/L	4.11 [3.47, 4.68]	4.14 [3.51, 4.70]	3.40 [2.64, 4.07]	0.705	<0.001
FT4 pmol/L	15.26 [13.30, 17.24]	15.25 [13.30, 17.22]	15.48 [13.28, 17.80]	0.066	0.014
FT3/FT4 ratio (× 100)	26.87 [22.67, 31.42]	27.05 [22.94, 31.57]	21.94 [17.06, 26.92]	0.160	<0.001
Tertiles of FT3/FT4 ratio				0.630	<0.001
T1	8,741 (33.33)	8,036 (32.05)	705 (61.30)		
T2	8,741 (33.33)	8,476 (33.81)	265 (23.04)		
T3	8,741 (33.33)	8,561 (34.14)	180 (15.65)		

SMD, standardized mean difference; TSH, Thyroid-Stimulating Hormone; FT3, Free Triiodothyronine; FT4, Free Thyroxine.

a 1990 missing values.

b 2065 missing values.

c 3273 missing values.

d 3273 missing values.

Regarding thyroid function parameters, survivors exhibited higher levels of TSH and FT3, as well as a higher FT3/FT4 ratio, while having lower FT4 levels compared with non-survivors (all *P* < 0.05).

The baseline data stratified by tertiles of FT3/FT4 ratio were presented in Supplementary Table S2.

### 3.2 Cox regression analysis between thyroid hormones and mortality

As shown in Table 2, in model 2, higher TSH levels were related to an increased risk of mortality (HR = 1.015, 95% CI: 1.010–1.020), whereas higher FT3 levels were significantly associated with lower mortality (HR = 0.865, 95% CI: 0.806–0.929). Additionally, a higher FT3/FT4 ratio (× 100) was also associated with reduced mortality risk (continuous: HR = 0.974, 95% CI: 0.964–0.983; T2 vs. T1: HR = 0.807, 95% CI: 0.688–0.947; T3 vs. T1: HR = 0.638, 95% CI: 0.529–0.770).

**Table 2:** Cox regression analysis between thyroid hormones and mortality.

Models	Events/*N*	Crude model	*P*	Events/N	Model 1	*P*	Events/*N*	Model 2	*P*
		HR (95% CI)			HR (95% CI)			HR (95% CI)	
Continuous variables									
TSH	1150/26221	1.017 (1.013, 1.022)	<0.001	1150/26221	1.018 (1.013, 1.023)	<0.001	990/22540	1.015 (1.010, 1.020)	<0.001
FT3	1150/26221	0.713 (0.671, 0.758)	<0.001	1150/26221	0.778 (0.730, 0.828)	<0.001	990/22540	0.865 (0.806, 0.929)	<0.001
FT4	1150/26221	1.019 (1.008, 1.031)	0.001	1150/26221	1.021 (1.007, 1.034)	0.003	990/22540	1.017 (1.002, 1.033)	0.022
FT3/FT4 ratio (× 100)	1150/26221	0.946 (0.938, 0.954)	<0.001	1150/26221	0.960 (0.952, 0.968)	<0.001	990/22540	0.974 (0.964, 0.983)	<0.001
FT3/FT4 ratio (× 100) tertiles									
T1	705/8,740	Ref.		705/8,740	Ref.		606/7538	Ref.	
T2	265/8,740	0.574 (0.497, 0.662)	<0.001	265/8,740	0.673 (0.583, 0.778)	<0.001	229/7492	0.807 (0.688, 0.947)	0.009
T3	180/8,741	0.425 (0.360, 0.502)	<0.001	180/8,741	0.528 (0.447, 0.625)	<0.001	155/7510	0.638 (0.529, 0.770)	<0.001

Model 1 adjusted for age and sex.

Model 2 adjusted for age, sex, marital status, admission method, admission vital signs, admission time period, comorbidities, coronary heart disease, diabetes mellitus, cancer, infection, stroke, heart failure, kidney disease, liver disease, ICU admission, smoking, and drinking.

The proportional hazards assumption was assessed using Schoenfeld residuals, and the results were presented in Supplementary Table S3. Although the global Schoenfeld residual test indicated violation of the proportional hazards assumption in the primary model, with age showing the strongest evidence of non-proportionality (Schoenfeld *P* = 3.2 × 10⁻⁸), no evidence of non-proportionality was observed for the FT3/FT4 ratio itself. In a sensitivity analysis stratified by age group, sex, coronary heart disease, and cancer, the association between the FT3/FT4 ratio and in-hospital mortality remained essentially unchanged (HR = 0.970, 95% CI: 0.961–0.980, *P* < 0.0001). In this stratified model, the proportional hazards assumption was not rejected for the FT3/FT4 ratio (*P* = 0.434) or globally (*P* = 0.089) **(**Supplementary Table S4**)**.

The unadjusted Kaplan–Meier survival curves **(**Figure 2**)** demonstrated differences in survival probability across FT3/FT4 ratio (× 100) tertiles (T1, T2, and T3) (log-rank *P* < 0.001).

**Figure 2:**
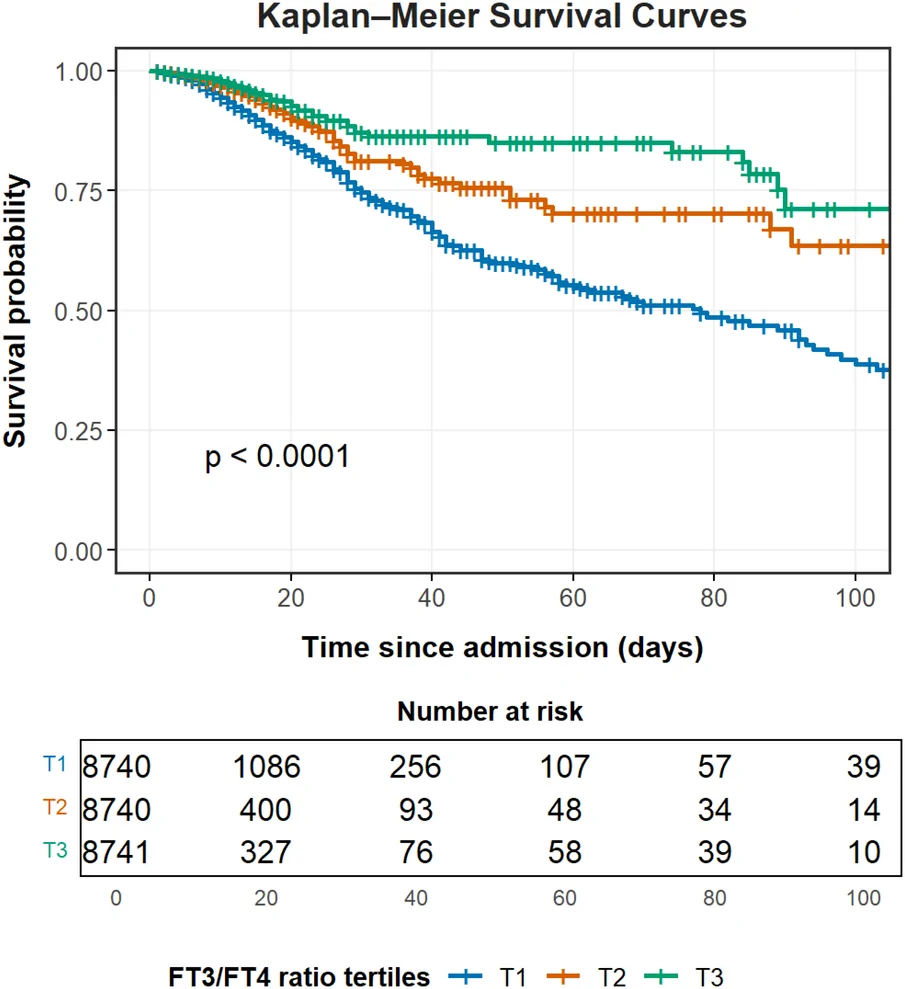
Kaplan–meier analysis of the survival probability in different groups of FT3/FT4 ratio. Unadjusted survival probabilities are shown for the three FT3/FT4 ratio tertiles (T1–T3), with the number at risk displayed below the curves. Differences between groups were assessed using the log-rank test. The x-axis is displayed through 100 days for visual clarity; all patients, including those with hospital stays longer than 100 days, were retained in the survival analysis.

### 3.3 The nonlinear relationship between thyroid hormones and mortality

RCS analyses showed significant evidence of non-linearity for TSH (*P* for non-linearity = 0.028), whereas no significant evidence of non-linearity was observed for FT3 (*P* = 0.767), FT4 (*P* = 0.958), or the FT3/FT4 ratio (*P* = 0.637). Figure 3 displays the adjusted RCS plots illustrating the continuous exposure–response relationships**.**

**Figure 3:**
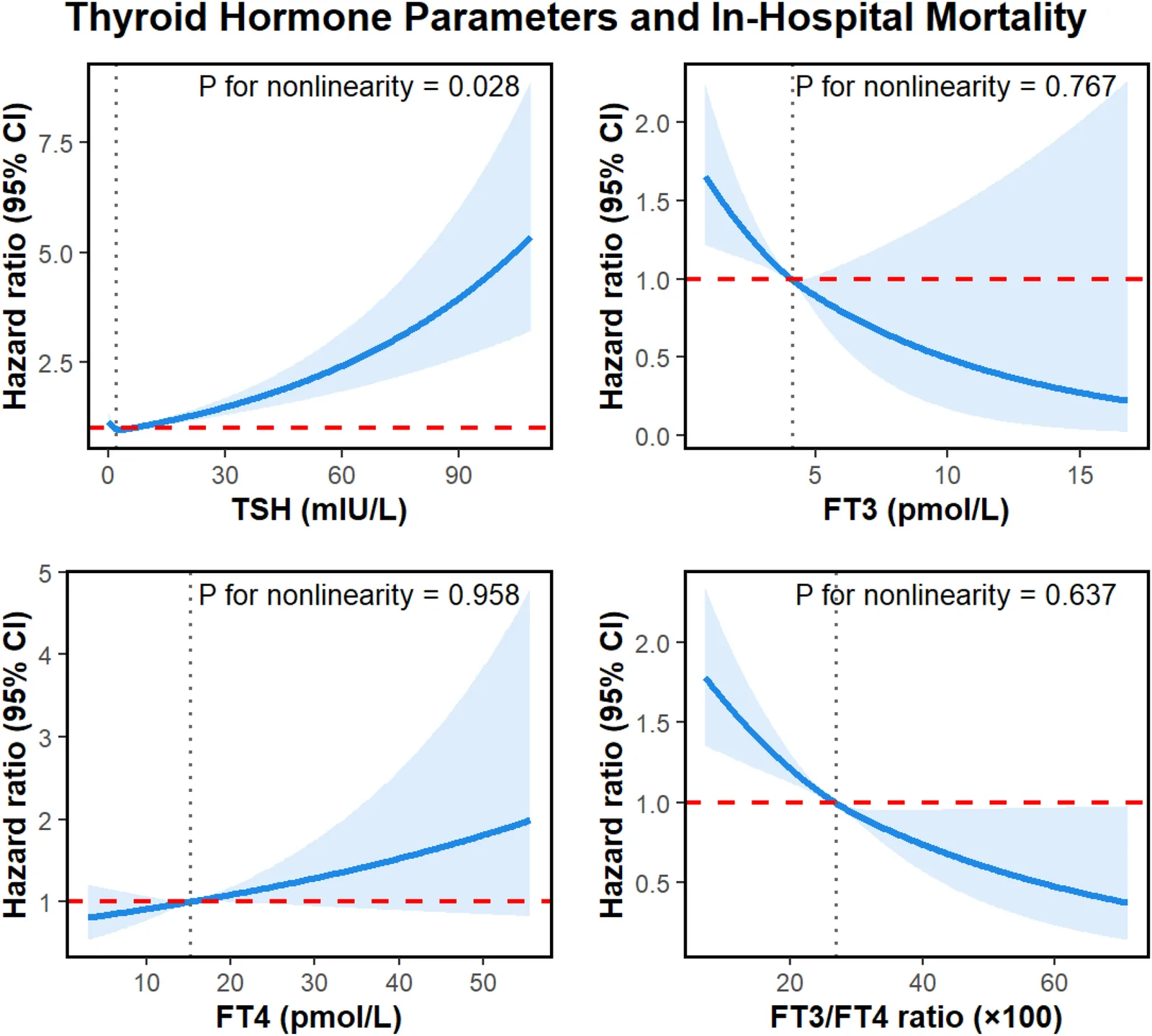
Restricted cubic spline (RCS) analysis was performed to explore the relationship between thyroid hormones levels and mortality probability. Adjusted for age, sex, marital status, admission method, admission vital signs, admission time period, comorbidities, coronary heart disease, diabetes mellitus, cancer, infection, stroke, heart failure, kidney disease, liver disease, ICU admission, smoking, and drinking. The median values were used as the reference (HR = 1): TSH, 1.82 mIU/L; FT3, 4.11 pmol/L; FT4, 15.27 pmol/L; and FT3/FT4 ratio×100, 26.85.

### 3.4 Sensitivity analysis

Sensitivity analyses were conducted after excluding participants in the lowest and highest 5% of the distributions separately for each TH parameter, resulting in sample sizes of 23,600 for TSH, 23,688 for FT3, 23,605 for FT4, and 23,599 for the FT3/FT4 ratio, respectively (Table 3). Sensitivity analyses generally supported the primary findings, particularly for FT3 and the FT3/FT4 ratio (× 100). The associations remained statistically significant for FT3, the continuous FT3/FT4 ratio (× 100), and the highest versus lowest FT3/FT4 tertile, whereas the associations for TSH, FT4, and the middle versus lowest FT3/FT4 tertile were attenuated and no longer statistically significant.

**Table 3:** Sensitivity analysis between thyroid hormones and mortality by cox regression.

Models	Events/*N*	Crude model	*P*	Events/N	Model 1	*P*	Events/N	Model 2	*P*
		HR (95% CI)			HR (95% CI)			HR (95% CI)	
Continuous variables									
TSH	885/23599	1.041 (0.990, 1.094)	0.115	885/23599	1.047 (0.996, 1.099)	0.069	750/20237	1.041 (0.987, 1.098)	0.138
FT3	902/23687	0.684 (0.624, 0.749)	<0.001	902/23687	0.767 (0.698, 0.842)	<0.001	772/20354	0.854 (0.768, 0.949)	0.003
FT4	951/23603	1.028 (1.002, 1.054)	0.035	951/23603	1.014 (0.989, 1.041)	0.276	816/20357	1.028 (0.999, 1.057)	0.055
FT3/FT4 ratio (× 100)	895/23597	0.946 (0.934, 0.958)	<0.001	895/23597	0.962 (0.950, 0.975)	<0.001	770/20317	0.973 (0.959, 0.987)	<0.001
FT3/FT4 ratio (× 100) tertiles									
T1	489/7,867	Ref.		489/7,867	Ref.		421/6,768	Ref.	
T2	234/7,864	0.642 (0.546, 0.754)	<0.001	234/7,864	0.758 (0.643, 0.892)	<0.001	203/6,736	0.845 (0.707, 1.010)	0.065
T3	172/7,866	0.468 (0.380, 0.576)	<0.001	172/7,866	0.591 (0.479, 0.930)	<0.001	146/6,813	0.665 (0.526, 0.841)	<0.001

Model 1 adjusted for age and sex.

Model 2 adjusted for age, sex, marital status, admission method, admission vital signs, admission time period, comorbidities, coronary heart disease, diabetes mellitus, cancer, infection, stroke, heart failure, kidney disease, liver disease, ICU admission, smoking, and drinking.

FT3 and the FT3/FT4 ratio (× 100) consistently showed significant inverse associations with mortality risk. Specifically, higher FT3 levels were associated with lower mortality risk (HR = 0.854, 95% CI: 0.768–0.949). Similarly, the FT3/FT4 ratio (× 100) remained significantly associated with reduced mortality risk in both continuous and tertile-based analyses (continuous: HR = 0.973, 95% CI: 0.959–0.987; T3 vs. T1: HR = 0.665, 95% CI: 0.526–0.841).

To further assess the potential impact of selection related to study inclusion, IPW analyses were performed using the entire source population. After primary IPW, all measured covariates achieved adequate balance, with the maximum absolute SMD decreasing from 0.153 before weighting to 0.019 after weighting and no covariates having an absolute SMD ≥ 0.10 **(**Supplementary Table S5**)**. The weighted analysis showed a consistent inverse association between the FT3/FT4 ratio (×100) and in-hospital mortality (HR = 0.954, 95% CI: 0.945–0.963, *P* < 0.0001). Compared with T1, the weighted HRs were 0.601 (95% CI: 0.515–0.701, *P* < 0.0001) for T2 and 0.490 (95% CI: 0.411–0.584, *P* < 0.0001) for T3 **(**Supplementary Table S6**)**.

The results were essentially unchanged in the expanded IPW analysis that additionally incorporated admission vital signs and their missingness indicators. All covariates remained adequately balanced after weighting (maximum absolute SMD = 0.069), and the corresponding HRs were 0.953 (95% CI: 0.943–0.962, *P* < 0.0001) for the continuous FT3/FT4 ratio (×100), 0.607 (95% CI: 0.517–0.712, *P* < 0.0001) for T2 versus T1, and 0.485 (95% CI: 0.404–0.582, *P* < 0.0001) for T3 versus T1 (Supplementary Table S6**)**.

### 3.5 Subgroup analysis between thyroid hormones and mortality

In the revised subgroup analyses using the continuous FT3/FT4 ratio, the inverse association with in-hospital mortality was generally observed across multiple patient subgroups (Supplementary Table S7). Formal interaction tests indicated evidence of heterogeneity according to sex, admission time period, coronary heart disease, diabetes mellitus, cancer, stroke, kidney disease, and smoking (all *P* for interaction <0.05). Notably, the FT3/FT4 ratio was inversely associated with in-hospital mortality among patients admitted during the day (HR = 0.970, 95% CI: 0.960–0.979), whereas no inverse association was observed among patients admitted at night (HR = 1.019, 95% CI: 0.988–1.051; *P* for interaction = 0.001). Evidence of heterogeneity was also observed according to kidney disease status (*P* for interaction = 0.020), with an inverse association among patients without kidney disease [HR = 0.968, 95% CI: 0.958–0.978] but a positive association among those with kidney disease [HR = 1.029, 95% CI: 1.005–1.053]. However, given the number of subgroup comparisons and the limited number of events in some strata, these findings should be considered exploratory and interpreted cautiously.

### 3.6 Exploratory association analysis

Exploratory analyses revealed that the FT3/FT4 ratio (× 100) was significantly associated with all four admission vital sign parameters after adjustment for confounders. Higher FT3/FT4 ratio was associated with lower heart rate (*β* = −0.242, *P* < 0.0001), lower systolic blood pressure (*β* = −0.918, *P* < 0.0001), and higher diastolic blood pressure (*β* = 0.071, *P* < 0.0001), while a modest association was observed with body temperature (*β* = −0.006, *P* = 0.011).

In Cox regression analyses, temperature was significantly associated with in-hospital mortality (HR = 1.002, 95% CI:1.000–1.004), whereas heart rate, systolic blood pressure, and diastolic blood pressure were not (Table 4).

**Table 4:** Exploratory association between FT3/FT4 ratio (× 100), admission vital signs, and in-hospital mortality.

Admission vital sign	Association with FT3/FT4 β (SE)	*P*	Association with mortality HR (95%CI)	*P*
Heart rate	−0.242 (0.015)	<0.001	0.945 (0.845, 1.058)	0.325
Temperature	−0.006 (0.002)	0.011	1.002 (1.000, 1.004)	0.041
Systolic pressure	−0.918 (0.225)	<0.001	0.998 (0.995, 1.002)	0.358
Diastolic pressure	0.071 (0.016)	<0.001	0.997 (0.991, 1.003)	0.387

Adjusted for age, sex, marital status, admission method, admission vital signs, admission time period, comorbidities, coronary heart disease, diabetes mellitus, cancer, infection, stroke, heart failure, kidney disease, liver disease, ICU admission, smoking, and drinking.

β represents the change in each admission vital sign (beats/min, °C, or mmHg, as appropriate) per one-unit increase in the FT3/FT4 ratio ×100.

## 4 Discussion

This study indicated that a 1-unit increase in TSH and FT4 levels was associated with a 1.5% and 1.7% increase in mortality risk, respectively, whereas a 1-unit increase in FT3 levels was associated with a 13.5% reduction in mortality risk. A 0.01-unit increase in the FT3/FT4 ratio was associated with a 2.6% reduction in mortality risk. Additionally, compared with the lowest FT3/FT4 ratio tertile, patients in the highest tertile exhibited a 36.2% lower mortality risk. Sensitivity analyses excluding extreme TH values generally supported the primary findings, particularly the inverse associations of FT3 and the FT3/FT4 ratio with in-hospital mortality. The inverse associations of FT3 and FT3/FT4 ratio with in-hospital mortality remained significant, with consistent results observed in both continuous and tertile-based analyses. Exploratory analyses showed that the FT3/FT4 ratio was associated with admission vital signs; however, only body temperature was independently associated with in-hospital mortality.

Previous studies have explored the association between thyroid status and mortality in specific populations, particularly among patients with renal or cardiovascular diseases. Thyroid-related parameters have been associated with CKD and with both all-cause and cardiovascular mortality among patients with CKD (10). In euthyroid patients undergoing percutaneous coronary intervention, low FT3/FT4 ratios were independently associated with increased long-term mortality risk (12) and were also linked to poorer clinical outcomes in CKD patients with crescentic pathology (9). FT4 levels and the FT3/FT4 ratio have also been identified as potential prognostic biomarkers in patients with acute aortic dissection (13), and a lower FT3/FT4 ratio was independently associated with increased long-term cardiac mortality (14). Studies in the U.S. population have reported that higher peripheral thyroid sensitivity, reflected by an elevated FT3/FT4 ratio (inflection point = 0.5), may be associated with reduced mortality risk in adults with metabolic syndrome (7, 15). Furthermore, critically ill Chinese patients with euthyroid sick syndrome (ESS)—characterized by reduced triiodothyronine (T3) and FT3levels, often exhibit poor clinical outcomes (16). Consistent with these previous findings, our study demonstrated an inverse association between the FT3/FT4 ratio and in-hospital mortality risk in hospitalized patients.

Although median TSH levels were slightly lower among non-survivors than survivors in the baseline comparison (1.73 vs. 1.81 mIU/L), the absolute difference was small. This apparent discrepancy may reflect the different information captured by descriptive comparisons and time-to-event analyses. While the baseline comparison summarizes differences in the distribution of TSH between outcome groups, Cox regression evaluates the association between continuous TSH levels and the hazard of in-hospital death over time. Notably, the positive association between TSH and mortality was already observed in the crude Cox model and remained directionally consistent after multivariable adjustment, indicating that the apparent discrepancy was not attributable to covariate adjustment. RCS analysis further suggested a nonlinear association between TSH and in-hospital mortality (*P* for non-linearity = 0.028), indicating that differences in median TSH levels alone may not fully characterize the association. In addition, the association was attenuated and no longer statistically significant after exclusion of extreme TSH values, suggesting that the TSH findings should be interpreted cautiously.

In exploratory analyses, the FT3/FT4 ratio was significantly associated with several admission vital signs, including heart rate, blood pressure, and temperature. Among these vital signs, only temperature remained independently associated with in-hospital mortality after multivariable adjustment, whereas heart rate, systolic blood pressure, and diastolic blood pressure were not. These findings suggest that FT3/FT4 ratio, admission vital signs, and mortality are associated; however, they do not support a potential mediating pathway. Given the concurrent measurement of THs and vital signs at admission, causal interpretation cannot be established. Our findings also differ from those reported in healthy or community-based populations, where higher FT3/FT4 ratios have been associated with increased heart rate and blood pressure (17, 18). This discrepancy may reflect the distinct physiological context of hospitalized patients, in whom TH levels are substantially influenced by acute illness, systemic inflammation, and metabolic stress and may therefore represent markers of disease severity rather than normal thyroid function.

Mechanistically, reduced FT3 levels may reflect impaired energy metabolism and altered substrate utilization, which could contribute to inadequate energy supply, oxidative stress, organ dysfunction, and increased mortality risk, particularly in critically ill or chronic disease populations (19). Furthermore, a low FT3/FT4 ratio has been associated with increased inflammatory markers, including C-reactive protein (CRP) and ferritin, potentially contributing to persistent inflammatory activation (20). Reduced FT3 levels may also be associated with enhanced sympathetic activity and renin-angiotensin-aldosterone system (RAAS) activation, which could further impair cardiac and renal function and worsen clinical prognosis (21, 22).

This study has several strengths. First, it included a relatively large sample of general inpatients from a large-scale hospital in China, providing sufficient statistical power to evaluate the associations between thyroid function parameters and in-hospital mortality. Second, comprehensive subgroup analyses were performed across demographic characteristics, clinical admission factors, comorbidities, and lifestyle factors, allowing exploration of the consistency of associations between thyroid hormone levels and mortality risk across different patient groups.

Nevertheless, several limitations should be acknowledged. First, because thyroid function testing was not routinely performed in all hospitalized patients, selection bias cannot be completely excluded. Comparison between included and excluded patients demonstrated generally small standardized mean differences for most baseline characteristics; however, excluded patients had a higher mortality rate than included patients (6.80% vs. 4.39%), suggesting potential differences between the analyzed and non-analyzed populations. Although the IPW analyses yielded consistent findings and achieved adequate balance in measured covariates, residual selection bias cannot be excluded because the weighting approach can account only for measured determinants of study inclusion. Therefore, prospective studies with systematic TH assessment are needed to validate these findings. Second, certain medications (e.g., thyroid hormone preparations, methimazole, propylthiouracil) may influence TH levels. However, medication information was unavailable, preventing adjustment for their potential effects. Third, despite adjustment for demographic characteristics, major comorbidities, primary admission diagnoses, and ICU admission as a proxy indicator of disease severity, residual confounding cannot be completely excluded. Several clinically relevant variables, including laboratory indicators (e.g., CRP, albumin, hemoglobin, renal and liver function), BMI, nutritional status, frailty, medications affecting thyroid function, and validated severity scores, were unavailable or contained substantial missing data and therefore could not be included in the primary analyses. Thus, the observed association between the FT3/FT4 ratio and in-hospital mortality should be interpreted as an independent association after adjustment for available confounders rather than evidence of causality. Future studies should incorporate a more comprehensive set of confounders to minimize residual confounding. Fourth, only in-hospital mortality was assessed, and whether the FT3/FT4 ratio is associated with long-term outcomes remains unclear. Fifth, the global Schoenfeld residual test indicated evidence of non-proportional hazards in the primary fully adjusted Cox model, although the FT3/FT4 ratio itself satisfied the proportional hazards assumption. To address non-proportionality among adjustment covariates, we conducted a sensitivity analysis using a Cox model stratified by age group, sex, coronary heart disease, and cancer. The association between the FT3/FT4 ratio and in-hospital mortality remained essentially unchanged in the stratified model, and the global proportional hazards test was no longer statistically significant. These findings support the robustness of the observed association to violations of the proportional hazards assumption in the primary model. Sixth, exploratory analyses of admission vital signs were intended to characterize potential associations only and should not be interpreted as evidence of causal pathways linking THs status to mortality. Finally, the generalizability of our findings may be limited because the study population was derived from a single hospital in Shenzhen. Future multicenter studies involving broader populations are warranted to confirm these findings.

## 5 Conclusion

In conclusion, this large-sample retrospective study identified an association between TH parameters, particularly the FT3/FT4 ratio, and in-hospital mortality among hospitalized patients. These findings may provide additional information for understanding TH status in hospitalized patients and support further investigation into their potential role in prognostic assessment.

## Data Availability

The datasets presented in this article are not readily available because of patient privacy protection and institutional ethical committee restrictions. Requests to access the datasets should be directed to the corresponding author.
