# Supplementary material for: Association between the FT3/FT4 ratio and in-hospital mortality among hospitalized patients undergoing thyroid function testing
Source: Front Med (Lausanne). 2026 Oct 27;13:1792939. doi: 10.3389/fmed.2026.1792939 (PMC13747953; doi:10.3389/fmed.2026.1792939)
Supplement: Supplementary file 1 [file Table1.docx]

Supplementary Material

## Supplementary Tables

**Supplementary Table S1.** baseline characteristics of included versus excluded patients

|  |  | **Patients** | |  |  |
| --- | --- | --- | --- | --- | --- |
| **Variables** | **Overall** | **Included** | **Excluded** | **SMD** | **P** |
| Num. | 66,210 | 26,223 | 39,987 |  |  |
| Age, years* | 67.00 [59.00, 75.00] | 67.00 [58.00, 75.00] | 68.00 [59.00, 76.00] | 0.008 | 0.009 |
| Sex (Male) | 35,615 (53.79) | 13,323 (50.81) | 22,292 (55.75) | 0.099 | <0.001 |
| Marital status |  |  |  | 0.073 | <0.001 |
| Divorced | 1,628 (2.46) | 731 (2.79) | 897 (2.24) |  |  |
| Married | 59,233 (89.46) | 23,487 (89.57) | 35,746 (89.39) |  |  |
| Separated | 3,505 (5.29) | 1,435 (5.47) | 2,070 (5.18) |  |  |
| Single | 1,168 (1.76) | 356 (1.36) | 812 (2.03) |  |  |
| Unknown | 676 (1.02) | 214 (0.38) | 462 (1.16) |  |  |
| Medical Insurance category |  |  |  | 0.138 | <0.001 |
| Urban Resident Insurance | 50,854 (76.81) | 21,046 (80.26) | 29,808 (74.54) |  |  |
| Rural Medical Insurance | 15,356 (23.19) | 5,177 (19.74) | 10,179 (25.46) |  |  |
| Admission method |  |  |  | 0.063 | <0.001 |
| Emergency | 10,207 (15.42) | 4,198 (16.01) | 6,009 (15.03) |  |  |
| Outpatient | 49,351 (74.54) | 19,673 (75.02) | 29,678 (74.22) |  |  |
| Others | 6,652 (10.05) | 2,352 (8.97) | 4,300 (10.75) |  |  |
| Admission vital signs |  |  |  |  |  |
| Body temperature (°C) † | 36.50 [36.30, 36.60] | 36.50 [36.30, 36.59] | 36.50 [36.40, 36.60] | 0.021 | <0.001 |
| Heart rate (bpm) ‡ | 78.00 [71.00, 86.00] | 77.00 [70.00, 85.00] | 78.00 [72.00, 86.00] | 0.098 | <0.001 |
| Systolic pressure (mmHg) § | 130.00 [118.00, 144.00] | 131.00 [118.00, 147.00] | 129.00 [117.00, 142.00] | 0.014 | <0.001 |
| Diastolic pressure (mmHg) \|\| | 78.00 [70.00, 85.00] | 78.00 [70.00, 86.00] | 78.00 [70.00, 85.00] | 0.022 | <0.001 |
| Admission time period |  |  |  | 0.051 | <0.001 |
| Nighttime | 3,091 (4.67) | 1,057 (4.03) | 2,034 (5.09) |  |  |
| Daytime | 63,119 (95.33) | 25,166 (95.97) | 37,953 (94.91) |  |  |
| Medical history |  |  |  |  |  |
| Hypertension | 31,507 (47.59) | 12,666 (48.30) | 18,841 (47.12) | 0.024 | 0.003 |
| Coronary heart disease | 7,675 (11.59) | 2,842 (10.84) | 4,833 (12.09) | 0.039 | <0.001 |
| Diabetes mellitus | 14,659 (22.14) | 5,911 (22.54) | 8,748 (21.88) | 0.016 | 0.045 |
| Cancer | 8704 (13.15) | 2127 (8.11) | 6577 (16.45) | <0.001 | 0.256 |
| Infection | 4101 (6.19) | 1047 (3.99) | 3054 (7.64) | <0.001 | 0.156 |
| Stroke | 8444 (12.75) | 3326 (12.68) | 5118 (12.80) | 0.671 | 0.003 |
| Heart failure | 646 (0.98) | 440 (1.68) | 206 (0.52) | <0.001 | 0.112 |
| Kidney diseases | 2075 (3.13) | 1521 (5.80) | 554 (1.39) | <0.001 | 0.239 |
| Liver diseases | 210 (0.32) | 59 (0.22) | 151 (0.38) | 0.001 | 0.028 |
| ICU admission | 8704 (13.15) | 795 (3.03) | 1755 (4.39) | <0.001 | 0.072 |
| Smoking ever | 14,607 (22.06) | 6,654 (25.37) | 7,953 (19.89) | 0.131 | <0.001 |
| Drinking | 8,621 (13.02) | 4,049 (15.44) | 4,572 (11.43) | 0.118 | <0.001 |
| Death | 3868 (5.84) | 1150 (4.39) | 2718 (6.80) | 0.105 | <0.001 |

SMD, standardized mean difference

* 37 missing values; † 5,985 missing values; ‡ 6108 missing values; § 8615 missing values; || 8615 missing values

**Supplementary Table S2.** Baseline data among all patients stratified by FT3/T4 ratio

|  |  | **Tertiles of FT3/FT4 ratio (× 100)** | | |  |
| --- | --- | --- | --- | --- | --- |
| **Variables** | **Overall** | **T1** | **T2** | **T3** | **P** |
| Num. | 26,223 | 8,741 | 8,741 | 8,741 |  |
| Age, years | 67.00 [58.00, 75.00] | 70.00 [61.00, 79.00] | 67.00 [58.00, 74.00] | 65.00 [57.00, 72.00] | <0.001 |
| Sex (Male) | 13,323 (50.81) | 4,306 (49.26) | 4,352 (49.79) | 4,665 (53.37) | <0.001 |
| Marital status |  |  |  |  | <0.001 |
| Divorced | 731 (2.79) | 256 (2.93) | 223 (2.55) | 252 (2.88) |  |
| Married | 23,487 (89.57) | 7,692 (88.00) | 7,883 (90.18) | 7,912 (90.52) |  |
| Separated | 1,435 (5.47) | 607 (6.94) | 484 (5.54) | 344 (3.94) |  |
| Single | 356 (1.36) | 153 (1.75) | 101 (1.16) | 102 (1.17) |  |
| Unknown | 214 (0.82) | 33 (0.38) | 50 (0.57) | 131 (1.50) |  |
| Medical Insurance category |  |  |  |  | 0.007 |
| Urban Resident Insurance | 21,046 (80.26) | 7,073 (80.92) | 7,052 (80.68) | 6,921 (79.18) |  |
| Rural Medical Insurance | 5,177 (19.74) | 1,668 (19.08) | 1,689 (19.32) | 1,820 (20.82) |  |
| Admission method |  |  |  |  | <0.001 |
| Emergency | 4,198 (16.01) | 2,213 (25.32) | 1,149 (13.14) | 836 (9.56) |  |
| Outpatient | 19,673 (75.02) | 5,953 (68.10) | 6,896 (78.89) | 6,824 (78.07) |  |
| Others | 2,352 (8.97) | 575 (6.58) | 696 (7.96) | 1,081 (12.37) |  |
| Admission vital signs |  |  |  |  |  |
| Body temperature (°C) * | 36.50 [36.30, 36.59] | 36.50 [36.30, 36.60] | 36.50 [36.30, 36.50] | 36.50 [36.30, 36.50] | <0.001 |
| Heart rate (bpm) † | 77.00 [70.00, 85.00] | 79.00 [71.00, 89.00] | 76.00 [69.00, 84.00] | 75.00 [68.00, 83.00] | <0.001 |
| Systolic pressure (mmHg) ‡ | 131.00 [118.00, 147.00] | 133.00 [118.00, 149.00] | 131.00 [118.00, 147.00] | 130.00 [118.00, 145.00] | <0.001 |
| Diastolic pressure (mmHg) § | 78.00 [70.00, 86.00] | 77.00 [70.00, 86.00] | 78.00 [71.00, 86.00] | 78.00 [71.00, 87.00] | <0.001 |
| Admission time period |  |  |  |  | <0.001 |
| Nighttime | 1057 (4.03) | 479 (5.48) | 263 (3.01) | 315 (3.60) |  |
| Daytime | 25,166 (95.97) | 8,262 (94.52) | 8,478 (96.99) | 8,426 (96.40) |  |
| Medical history |  |  |  |  |  |
| Hypertension | 12,666 (48.30) | 4,841 (55.38) | 4,076 (46.63) | 3,749 (42.89) | <0.001 |
| Coronary heart disease | 2,842 (10.84) | 1,205 (13.79) | 842 (9.63) | 795 (9.10) | <0.001 |
| Diabetes mellitus | 5,911 (22.54) | 2,596 (29.70) | 1,880 (21.51) | 1,435 (16.42) | <0.001 |
| Cancer | 2127 (8.11) | 715 (8.18) | 589 (6.74) | 823 (9.42) | <0.001 |
| Infection | 1047 (3.99) | 678 (7.76) | 189 (2.16) | 180 (2.06) | <0.001 |
| Stroke | 3326 (12.68) | 1217 (13.92) | 1086 (12.42) | 1023 (11.70) | <0.001 |
| Heart failure | 440 (1.68) | 286 (3.27) | 104 (1.19) | 50 (0.57) | <0.001 |
| Kidney diseases | 1521 (5.80) | 754 (8.63) | 438 (5.01) | 329 (3.76) | <0.001 |
| Liver diseases | 59 (0.22) | 19 (0.22) | 14 (0.16) | 26 (0.30) | 0.157 |
| ICU admission | 795 (3.03) | 540 (6.18) | 132 (1.51) | 123 (1.41) | <0.001 |
| Smoking ever | 6,654 (25.37) | 2,127 (24.33) | 2,231 (25.52) | 2,296 (26.27) | 0.012 |
| Drinking | 4,049 (15.44) | 1,197 (13.69) | 1,374 (15.72) | 1,478 (16.91) | <0.001 |
| TSH | 1.80 [1.10, 2.91] | 1.54 [0.86, 2.60] | 1.85 [1.18, 2.89] | 2.01 [1.27, 3.24] | <0.001 |
| FT3 | 4.11 [3.47, 4.68] | 3.43 [2.86, 3.91] | 4.19 [3.76, 4.61] | 4.67 [4.19, 5.10] | <0.001 |
| FT4 | 15.26 [13.30, 17.24] | 17.03 [14.97, 19.14] | 15.58 [13.99, 17.09] | 13.57 [11.94, 15.07] | <0.001 |
| FT3/FT4 ratio (× 100) | 26.87 [22.67, 31.42] | 20.71 [17.87, 22.67] | 26.87 [25.63, 28.25] | 33.49 [31.42, 36.74] | <0.001 |

TSH, Thyroid-Stimulating Hormone; FT3, Free Triiodothyronine; FT4, Free Thyroxine

* 1990 missing values; † 2065 missing values; ‡ 3273 missing values; § 3273 missing values

**Supplementary Table S3.** Test of proportional hazards assumption using Schoenfeld residuals for Cox regression models

| Variables | TSH model | FT3 model | FT4 model | FT3/FT4 ratio (× 100) model |
| --- | --- | --- | --- | --- |
| Age, years | 1.2e-08 | 2.3e-08 | 2.1e-08 | 3.2e-08 |
| Sex (Male) | 0.042 | 0.047 | 0.049 | 0.041 |
| Marital status | 0.668 | 0.686 | 0.634 | 0.662 |
| Admission method | 0.174 | 0.236 | 0.139 | 0.206 |
| Admission time period | 0.625 | 0.715 | 0.696 | 0.673 |
| Body temperature (°C) | 0.496 | 0.600 | 0.430 | 0.583 |
| Heart rate (bpm) | 0.063 | 0.086 | 0.066 | 0.074 |
| Systolic pressure (mmHg) | 0.705 | 0.929 | 0.760 | 0.969 |
| Diastolic pressure (mmHg) | 0.030 | 0.061 | 0.023 | 0.063 |
| Hypertension | 0.072 | 0.101 | 0.055 | 0.065 |
| Coronary heart disease | 0.003 | 0.004 | 0.003 | 0.003 |
| Diabetes mellitus | 0.448 | 0.703 | 0.586 | 0.691 |
| Cancer | 0.006 | 0.007 | 0.008 | 0.004 |
| Infection | 0.518 | 0.526 | 0.486 | 0.456 |
| Stroke | 0.284 | 0.346 | 0.292 | 0.312 |
| Heart failure | 0.487 | 0.561 | 0.515 | 0.529 |
| Kidney diseases | 0.944 | 0.864 | 0.926 | 0.960 |
| Liver diseases | 0.276 | 0.268 | 0.284 | 0.305 |
| ICU admission | 0.688 | 0.657 | 0.772 | 0.633 |
| Smoking ever | 0.384 | 0.291 | 0.271 | 0.154 |
| Drinking | 0.001 | 0.005 | 0.003 | 0.008 |
| TSH | 0.467 | NA | NA | NA |
| FT3 | NA | 0.213 | NA | NA |
| FT4 | NA | NA | 0.570 | NA |
| FT3/FT4 ratio (× 100) | NA | NA | NA | 0.229 |
| Global | <0.001 | <0.001 | <0.001 | <0.001 |

**Supplementary Table S4.** Sensitivity analysis accounting for nonproportional hazards

| **Analysis** | **Events/N** | **HR (95% CI)** | **P** | **PH P for FT3/FT4** | **Global PH P** |
| --- | --- | --- | --- | --- | --- |
| Primary model 2* | 990/22,540 | 0.974 (0.964, 0.983) | <0.001 | 0.229 | <0.001 |
| Stratified Cox model† | 990/22,540 | 0.970 (0.961–0.980) | <0.001 | 0.434 | 0.089 |

* Model 2 adjusted for age, sex, marital status, admission method, admission vital signs, admission time period, comorbidities, coronary heart disease, diabetes mellitus, cancer, infection, stroke, heart failure, kidney disease, liver disease, ICU admission, smoking, and drinking

† Stratified by age group, sex, coronary heart disease, and cancer.

**Supplementary Table S5.** Covariate balance before and after IPW

| Covariate | Before weighting | After Primary IPW | After Expanded IPW |
| --- | --- | --- | --- |
| Age | 0.002 | 0.008 | 0.011 |
| Sex (male) | −0.060 | 0.003 | 0.005 |
| Marital status (married) | 0.003 | −0.005 | −0.009 |
| Marital status (separated) | 0.008 | 0.004 | 0.005 |
| Marital status (single) | −0.031 | 0.001 | 0.004 |
| Marital status (unknown) | −0.020 | 0.003 | 0.005 |
| Admission method (outpatient) | 0.011 | −0.009 | −0.019 |
| Admission method (others) | −0.036 | 0.002 | 0.005 |
| Hypertension | 0.014 | −0.005 | −0.003 |
| Coronary heart disease | −0.024 | 0.003 | 0.006 |
| Diabetes mellitus | 0.010 | 0.003 | 0.005 |
| Cancer | −0.149 | 0.011 | 0.007 |
| Infection | −0.091 | 0.009 | 0.033 |
| Stroke | −0.002 | 0.008 | 0.007 |
| Heart failure | 0.071 | 0 | −0.001 |
| Kidney diseases | 0.153 | 0 | −0.001 |
| Liver diseases | −0.016 | 0.002 | 0.005 |
| ICU admission | −0.043 | 0.019 | 0.036 |
| Smoking ever | 0.080 | −0.006 | −0.006 |
| Drinking | 0.072 | −0.001 | −0.001 |
| Body temperature | −0.089 | NA | 0.069 |
| Body temperature missingness | −0.051 | NA | -0.001 |
| Heart rate | −0.051 | NA | 0.037 |
| Heart rate missingness | −0.046 | NA | -0.002 |
| Systolic pressure | 0.073 | NA | -0.01 |
| Systolic pressure missingness | −0.016 | NA | -0.003 |
| Diastolic pressure | 0.037 | NA | -0.009 |
| Diastolic pressure missingness | −0.016 | NA | -0.002 |

**Supplementary Table S6**. Inverse-probability-of-selection-weighted Cox regression analysis of the association between the FT3/FT4 ratio and in-hospital mortality

|  |  | | **Primary IPW** |  |  | **Expanded IPW** |  |
| --- | --- | --- | --- | --- | --- | --- | --- |
| **Models** | **Events/N** | | **HR (95% CI)** | **P** | **Events/N** | **HR (95% CI)** | **P** |
| Continuous variables |  | |  |  |  |  |  |
| TSH | 1150/26,221 | | 1.020 (1.016, 1.024) | <0.001 | 1150/26,221 | 1.020 (1.016, 1.024) | <0.001 |
| FT3 | 1150/26,221 | | 0.727 (0.679, 0.779) | <0.001 | 1150/26,221 | 0.722 (0.672, 0.775) | <0.001 |
| FT4 | 1150/26,221 | | 1.012 (0.999, 1.025) | <0.001 | 1150/26,221 | 1.012 (0.998, 1.025) | <0.001 |
| FT3/FT4 ratio (× 100) | 1150/26,221 | | 0.954 (0.945, 0.963) | <0.001 | 1150/26,221 | 0.953 (0.943–0.962) | <0.001 |
| FT3/FT4 ratio (× 100) tertiles | |  |  |  |  |  |  |
| T1 | 705/8,740 | | Ref. |  | 705/8,740 | Ref. |  |
| T2 | 265/8,740 | | 0.601 (0.515, 0.701) | <0.001 | 265/8,740 | 0.607 (0.517–0.712) | <0.001 |
| T3 | 180/8,741 | | 0.490 (0.411, 0.584) | <0.001 | 180/8,741 | 0.485 (0.404–0.582) | <0.001 |

Primary IPW was based on a selection model including age, sex, marital status, admission approach, hypertension, coronary heart disease, diabetes mellitus, cancer, infection, stroke, heart failure, kidney disease, liver disease, intensive care unit admission, smoking, and drinking.

Expanded IPW additionally included temperature, heart rate, systolic blood pressure, and diastolic blood pressure. Missing vital-sign values were median-imputed, and corresponding missingness indicators were included in the expanded selection model.

**Supplementary Table** **S7.** Subgroup analysis between FT3/FT4 ratio and mortality

| **Variables** | **Events/N** | **HR (95% CI)** | **P** | **P for interaction** |
| --- | --- | --- | --- | --- |
| **Age** |  |  |  | 0.106 |
| <60 | 105/6,079 | 0.959 (0.929, 0.989) | 0.009 |  |
| ≥60 | 885/16,461 | 0.970 (0.960, 0.979) | <0.001 |  |
| **Sex** |  |  |  | <0.001 |
| Female | 416/11,155 | 0.955 (0.941, 0.970) | <0.001 |  |
| Male | 574/11,385 | 0.986 (0.974, 0.998) | 0.018 |  |
| **Marital status** |  |  |  | 0.357 |
| Married | 880/20,243 | 0.975 (0.965, 0.985) | <0.001 |  |
| Divorced | 92/1,225 | 0.981 (0.950, 1.013) | 0.234 |  |
| Separated | 11/636 | 0.940 (0.847, 1.043) | 0.244 |  |
| **Admission method** |  |  |  | 0.321 |
| Outpatient | 643/17,080 | 0.974 (0.962, 0.985) | <0.001 |  |
| Emergency | 217/3,384 | 0.970 (0.949, 0.993) | 0.010 |  |
| **Admission time period** |  |  |  | 0.001 |
| Day | 926/21,681 | 0.970 (0.960, 0.979) | <0.001 |  |
| Night | 64/859 | 1.019 (0.988, 1.051) | 0.229 |  |
| **Hypertension** |  |  |  | 0.467 |
| Yes | 591/10,906 | 0.980 (0.968, 0.993) | 0.002 |  |
| No | 399/11,634 | 0.971 (0.957, 0.985) | <0.001 |  |
| **Coronary heart disease** |  |  |  | 0.044 |
| Yes | 201/2,405 | 0.991 (0.971, 1.012) | 0.414 |  |
| No | 789/20,135 | 0.971 (0.960, 0.981) | <0.001 |  |
| **Diabetes mellitus** |  |  |  | 0.013 |
| Yes | 271/5,112 | 1.001 (0.982, 1.020) | 0.939 |  |
| No | 719/17,428 | 0.966 (0.955, 0.976) | <0.001 |  |
| **Cancer** |  |  |  | <0.001 |
| Yes | 218/1,973 | 0.982 (0.963, 1.000) | 0.052 |  |
| No | 772/20,567 | 0.968 (0.957, 0.979) | <0.001 |  |
| **Infection** |  |  |  | 0.995 |
| Yes | 149/882 | 0.957 (0.931, 0.984) | 0.002 |  |
| No | 841/21,658 | 0.977 (0.967, 0.987) | <0.001 |  |
| **Stroke** |  |  |  | 0.038 |
| Yes | 50/2,639 | 0.970 (0.927, 1.015) | 0.192 |  |
| No | 940/19,901 | 0.975 (0.966, 0.985) | <0.001 |  |
| **Heart failure** |  |  |  | 0.840 |
| Yes | 63/380 | 1.034 (0.990, 1.081) | 0.135 |  |
| No | 927/22,160 | 0.973 (0.964, 0.983) | <0.001 |  |
| **Kidney diseases** |  |  |  | 0.020 |
| Yes | 133/1,385 | 1.029 (1.005, 1.053) | 0.885 |  |
| No | 857/21,155 | 0.968 (0.958, 0.978) | 0.125 |  |
| **Liver diseases** |  |  |  | 0.353 |
| Yes | 2/51 | NE* | — |  |
| No | 988/22,489 | 0.974 (0.965, 0.983) | <0.001 |  |
| **ICU admission** |  |  |  | 0.074 |
| Yes | 173/716 | 0.984 (0.961, 1.007) | 0.170 |  |
| No | 817/21,824 | 0.976 (0.966, 0.986) | <0.001 |  |
| **Smoking ever** |  |  |  | 0.029 |
| Yes | 250/5,700 | 0.993 (0.975, 1.010) | 0.419 |  |
| No | 740/16,840 | 0.966 (0.955, 0.977) | <0.001 |  |
| **Drinking** |  |  |  | 0.452 |
| Yes | 131/3,446 | 0.994 (0.969, 1.020) | 0.656 |  |
| No | 859/19,094 | 0.971 (0.961, 0.981) | <0.001 |  |

Adjusted for age, sex, marital status, admission method, admission vital signs, admission time period, comorbidities, coronary heart disease, diabetes mellitus, cancer, infection, stroke, heart failure, kidney disease, liver disease, ICU admission, smoking, and drinking

* Not estimable because of the very small number of events.
